# Electromagnetic field in human sperm cryopreservation improves fertilizing potential of thawed sperm through physicochemical modification of water molecules in freezing medium

**DOI:** 10.1371/journal.pone.0221976

**Published:** 2019-09-05

**Authors:** Dariush Gholami, Seyed Mahmood Ghaffari, Gholamhossein Riazi, Rouhollah Fathi, James Benson, Abdolhossein Shahverdi, Mohsen Sharafi

**Affiliations:** 1 Institute of Biochemistry and Biophysics (IBB), University of Tehran, Tehran, Iran; 2 Department of Embryology at Reproduction Biomedicine Research Center, Royan Institute for Reproductive Biomedicine, ACER, Tehran, Iran; 3 Department of Biology, University of Saskatchewan, Saskatoon, Canada; 4 Reproductive Epidemiology Research Center, Royan Institute for Reproductive Biomedicine, ACECR, Tehran, Iran; 5 Department of Poultry Sciences, Faculty of Agriculture, Tarbiat Modares University, Tehran, Iran; University Hospital of Münster, GERMANY

## Abstract

Physicochemical properties of water molecules as the main compositions of the freezing media can be affected by the electromagnetic fled. The purpose of this study was to apply extremely low repetition rate electromagnetic fields (ELEFs) to change the molecular network of water molecules existing in freezing media used for human sperm cryopreservation. First, different time periods and pulsed electromagnetic fields were used to evaluate the physiochemical properties of water. The lowest rate of cluster size, surface tension, viscosity, and density was observed for water samples exposed to 1000 Hz ELEF for 60 min (P < 0.05) that could be results in small ice crystal formation. Therefore, this treatment was selected for further evaluations in human sperm freezing because there was minimal probability of amorphous ice crystallization in this group. To assess fertilizing potential, human semen samples were subjected to ELEF (1000 Hz) water-made freezing medium and cryopreserved. The highest percentage of total motility, progressive motility, viability, membrane integrity, mitochondrial membrane potential, DNA integrity, and TAC were obtained in frozen ELEF as compared to other groups. The percentage of viable spermatozoa (Annexin V^-^/PI^-^) in frozen ELEF was significantly higher than in frozen control. The level of ROS was significantly lower in frozen ELEF when compared to frozen control. It can be concluded that the modification of physicochemical properties of water existing in cryopreservation media by ELEF is a suitable strategy to improve the outcome of cryopreservation.

## Introduction

The cryopreservation of living cells and tissues in the world of biotechnology has been developed tremendously because this process allows the recovery of large populations of eukaryotic and prokaryotic cells at very low temperatures [1, 2]. Human semen cryopreservation is one of those beneficial approaches that allows the storage of sperm and thus conserves their sperm quality [3].

Despite several advantages of this strategy, structural and biochemical damages caused by this process to sperm are the main challenge and drawback [4, 5] which lead to reduce the fertilizing potential of thawed sperm. This phenomenon is mostly related to sudden disruption of water molecule structures in cryopreservation media that can induce the production of ROS which, in turn, attacks to the sperm membrane and damage the sperm organelles resulting in sperm death [6, 7]. Water can form 16 different ice crystal structures during the freezing process; among these 16 structures, the hexagonal structure causes the greatest damage to cells during the cryopreservation [8].

Based on biophysical properties of water, a novel strategy developed to disrupt the regular network of water molecules in cryopreservation media with the purpose of reduction of hexagonal crystal structure formation and ROS production. It has been reported that physicochemical characteristics of water, including surface tension, viscosity, density, and light distribution characteristics can affected by the external factors such as different solutes, electrical, magnetic and electromagnetic fields, and temperature [9–12].

In an earlier study, electromagnetic waves were used to break up the regular structure of the water molecules network [13] that could help the formation of small-size water clusters which is an accurate way to detect changes in the structures and mechanisms of transition from single to bulk monomers [14]. In the present study, ELEFs were applied to disrupt the network of water molecules in order to affect the size of local clusters formed by water and subsequently reduction of hexagonal ice crystal formation during cryopreservation. It has been reported that the small ice crystals were not necessarily lethal for cells, while large ice crystals were associated with lethal damage during rapid cooling rate of freezing [15, 16]. Moreover, most of the dead cells after thawing have large ice crystal with size of more than 2–3 μm [17]. In the low temperatures, when water cluster grows in the range 300 molecules, the hexagonal structure of the ice crystal formed in the cluster core. It has been postulated that ELEFs can change this event and inhibit the size of clusters that can lead to small ice crystal formation.

In the other hand, one of the important steps in the formation of ice crystals is the ice nucleation that, when this nucleus occurs earlier, the ice crystal formed is larger. Ice nucleation is depends on the supercooling degree of water, and any factor that changes the degree of supercooling will affect the induction or containment of ice nucleation, and subsequently affect the ice crystal morphology such as shape and size distributions [18, 19]. The electromagnetic fields prohibit ice nucleation due to reciprocating alignment of the water molecules and subsequently increase the degree of supercooling that lead to formation of smaller ice crystals [20].

Therefore, the objective of the present study was to disrupt the regular network of the water molecules using electromagnetic fields to induce changes in the water and alters the shape and size distributions of ice crystals during the human semen cryopreservation. According to the physicochemical characteristics of water samples treated with ELEFs, the optimum water sample was selected to prepare the human sperm cryopreservation media. Finally, after cryopreservation of sperm with the media prepared with the optimum water, several indicators of sperm quality such as motion characteristics, viability, apoptotic status, membrane integrity, acrosome integrity, mitochondria activity, DNA fragmentation, ROS, and TAC were assessed.

## Material and methods

### Chemicals and ethics

All chemicals used in this study were purchased from Sigma (St. Louis, MO, USA) unless mentioned otherwise.

For this study, an approval was obtained from the Research Ethics Committee of Royan Institute, Tehran, Iran, (http://ethics.research.ac.ir/IR.ACECR.ROYAN.REC.1397.033), and it was conducted according to the ethical guidelines of the Helsinki Declaration. All the experiments were performed according to the national and international guidelines. All individuals were referred to the Royan Institute in Iran, and informed about the study procedures and the usage of their clinical and biological data for the research purposes. Written informed consent for the study participants was also obtained.

### Exposure of water to ELEFs and physicochemical analysis

#### Experimental design

Glass cells containing double distilled water (prepared by Double Distiller GFL model 2108, Burgwedel, Germany) were placed in one of the two insulated cages and exposed to ELEFs in a factorial design study (two factors: time periods and repetition rates). Five levels of time periods (0, 15, 30, 45 and 60 min) and 10 levels of repetition rates (100, 200, 300, 400, 500, 600, 700, 800, 900 and 1000 Hz) were applied in a factorial design to electeromagnetized water. Electromagnetic field generator used in this study was a Helmholtz coil (The radius of each coil 35 mm, 70 mm high, copper wire, 1000 turns/m, the diameter of the wire in each coil 1.7 mm, self-inductance L = 3 mH, ohmic resistance = 3 Ω). The RMS magnetic field at the center of the Helmholtz coil was almost 1.5 mT and the maximum induced electric field was 4.1 mV/m, according to Faraday's law. Untreated glass cells (as controls) were placed in the other insulated cage in which the conditions were the same as for the exposed groups but without ELEF. After exposure to ELEF, treated and untreated glass cells were transferred into separate storage boxes. The temperature was monitored using a chromel-alumel thermocouple, during the electromagnetic treatment and Δθ was < 0.01°C.

#### Cluster size analysis

The cluster diameter (d) was obtained based on the Stokes-Einstein relation:

$$D=k_{B}T/3\pi \eta (t)d$$ (1)

where k_B_ is Boltzmann constant (1.38054×10^−16^ ergs/deg), T is the absolute temperature in °K, η(t) is the viscosity of the solution (in centipoise) and D is the diffusion coefficient [21]. The sample was loaded into cuvette chambers and the cluster size was determined by measuring the cluster diameter of water by the DLS (Brookhaven Instruments Corporation, USA).

#### Dynamic surface tension and viscosity

In this study, water surface tension force was measured using KRÜSS K100 Tensiometer (Germany) with the accuracy of ± 0.01, resolution of 0.0001 mN/m, sample vessel size of 50, 70 and 100 mm, humidity of 27% and Power Supply of 40 W. Rheometer (Physica MCR302, Paar Physica, Austria) was used to measure the viscosity of treated and untreated water samples at room temperature.

#### Water density

Water density was assessed by Densitometer (DTM-500 Black-White model, NDT Supply, USA) with aperture size diameter 2 mm, power 220V ± 10%, power dissipation 25 W, repeatability ± 0.01 D and an accuracy of ± 0.02. To prevent the formation of bubbles in the electromagnetic water during the experiment, water samples were degassed before the use. The measurements were done at the room temperature (298° K).

#### Water memory measurement

The water memory for physicochemical properties of the network of water molecules was performed at 0, 1, 6, 12, 24, 48, and 96 hours after applying the 1000 Hz ELEF for 60 min as the optimal electromagnetic field. In this experiment, distilled water without being affected by ELEF was used as a control (0 Hz).

### Human semen cryopreservation with ELEF water made freezing medium

#### Freezing media preparation

According to physicochemical data of water samples, the optimum sample was selected to prepare the freezing media which was consisted of 1000 Hz ELEF-treated water supplemented with TES, tris, glycerol (10%), DMSO (2%) and soybean lecithin (1%; P3556, purity > 99%, Sigma). Freezing media were set at 325 mOsm and adjusted to pH 7.5, before adding lecithin. Lecithin was added to TEST buffer and the mixture was centrifuged at 1,000 g for 20 min. The supernatant was then filtered through 0.45 μm Acrodisc syringe filters (Merck Millipore Company, U.S). Finally, glycerol and DMSO were added to the TEST lecithin medium.

#### Semen collection, processing, cryopreservation, and thawing

Semen samples were obtained from 25 healthy men (with normal morphology and having 4% or more normal forms, motility over 40%, and sperm concentration of over 20×10^6^/ml) according to WHO criteria [22], with 3–5 days of sexual abstinence. After primary evaluation of semen, the specimens were equally divided into three sets of aliquots and processed as following experimental groups; 1) the aliquot that was not cryopreserved and used as fresh control, 2) the aliquot that was cryopreserved with control freezing medium without ELEF-treated water (frozen control) and 3) the aliquot that was cryopreserved with freezing medium containing ELEF-treated water (frozen ELEF).

For cryopreservation, After liquefaction of the samples at 37°C for 30 min, rapid freezing was performed using a method previously described by Jeyendran et al. [23] with some modifications. Briefly, semen samples were slowly mixed in a dropwise manner with an equal volume of each medium. The mixture was then loaded into labeled 0.5 mL French straws and placed in liquid nitrogen vapor (-80°C) for 15 min and subsequently frozen in liquid nitrogen until the analysis.

According to the method of Jeyendran et al., frozen-thawed specimens were diluted four-fold with Tyrode’s salt solution. The diluted samples were centrifuged at 800 g for 4 min at the room temperature. Then, the sperm pellets were gently re-suspended in 0.5 mL of Tyrode’s salt solution. The post-thaw sperm suspension was incubated at 37°C in 5% CO2 for 20 min followed by sperm analysis.

#### Motion characteristics

Motion characteristics of thawed sperm were determined using a computer-assisted sperm analyzer (CASA, Version 5.1; Microptic, Barcelona, Spain). For this purpose, 5 μL of sperm suspension was loaded onto a pre-warmed 20 μm chamber (Leja 4, Leja Products Luzernestraat B.V., Holland). A minimum of 5 fields per sample was evaluated (a minimum of 300 was counted for each sample) and the following parameters were analyzed; motility (%), progressive motility (%),VCL (μm/sec), VSL (μm/sec), VAP (μm/sec), LIN (%),STR (%), ALH (μm), and BCF (Hz).

#### Viability

For the evaluation of viability, eosin-nigrosin staining was applied as described by Bjorndahl et al. [24]. The eosin-nigrosin staining solution (50 μl) was added to a 50 μl of the sperm solution. The suspension was then incubated for 30 sec at 25°C. A smear of the mixture was stained on a microscope slide and observed under the light microscopy at 1000× magnification. White sperm was considered as the live and those with pink or red coloration (stained) were considered as the dead cells. The survival average percentage was measured by counting at least 200 cells per slide.

#### Membrane integrity

HOST was carried out according to WHO criteria [22]. Briefly, 0.735 gr of sodium citrate dihydrate and 1.351gr of D-fructose were added in 100 ml of purified water to obtain a 150 mOsmol of the hypo-osmotic swelling solution. A 1:2 ratio of semen was mixed with the hypo-osmotic solution at 37°C for 30 min and then the different HOST sperm-tail patterns were counted using the light microscope at 100× magnification. Then, 300 sperm was randomly assessed to determine the percentage of swollen and non-swollen tails visualized under a phase-contrast microscope (400× magnifications, CKX41, Olympus, Tokyo, Japan).

#### Abnormal morphology

To assess the abnormal morphology, each sample (20 μl) was placed on a slide and air dried. The smears were manually stained by Papanicolaou staining. Two hundred sperm was counted for each specimen and percentages of acrosome and head abnormalities were determined by light microscopy at 100× magnification.

#### Lipid peroxidation

Lipid peroxidation was measured based on the MDA level using TBA method with a slight modifications [25, 26]. The MDA concentration was assessed both in the seminal plasma and sperm. Specimens from each group were centrifuged at 1500 g for 5 min and then seminal plasma and sperm pellet were separated to measure the level of MDA.

For the measurement of MDA level in seminal plasma, 1:2 ratio of TBA was added to seminal plasma and incubated immediately at 95°C for 30 min and then allowed to cool on ice for 5 min. Afterward, the specimens were centrifuged at 1500 g for 5 min and the supernatant absorbance determined by spectrophotometer at the wavelength of 535 nm.

For the measurement of the MDA concentration in sperm, the concentration of 20×10^6^ sperm/ml was adjusted by addition of PBS. The sperm was incubated with 250 μl of 2.5 mM ferrous sulfate and 250 μl of 12.5 mM sodium ascorbate in a water bath at 37°C for 60 min. 500 μl of 40% ice-cold TCA was added to precipitate the proteins and the samples were then centrifuged at 1600 g for 12 min. 500 μL of 2% TBA and 0.2 N of NaOH were added to the 1mL of supernatant and solution boiled at 100°C for 10 min. The samples were cooled on ice for 10 min and the absorbance was measured by spectrophotometer at the wavelength of 535 nm. The concentration of MDA was determined by the specific absorbance coefficient (1.5×105 mol^-1^.cm^-1^). The following equation used to calculate the MDA (Banday, Lone et al. 2017):

$$MDA\;level\;(\frac{nmol}{ml})=(\frac{OD\times 10^{6}}{1.56\times 10^{5}})\times (\frac{Total\;volume}{Sample\;volume})$$ (2)

#### ROS by chemiluminescence

The semen samples were liquefied in an incubator at 37°C for 20 min. Samples are centrifuged at 300 g for 7 min, and the pellet is suspended in 3 ml of Dulbecco’s PBS and centrifuged again at 300 g for 7 min. The sperm concentration is adjusted to 20×10^6^ sperm/ml and ROS measurement was then processed. The extracellular ROS levels were assessed by the chemiluminescence assay using luminol (5-amino-2, 3-dihydro-1, 4-phthalazinedione; Sigma, USA) as a probe. For the evaluating ROS in semen, 10 μl luminol working solution (5 mM luminol prepared in DMSO) was added to 400 μl liquefied sperm suspension (20×10^6^ sperm/ml) and mixed gently.

Positive controls were prepared by 395 μl PBS, 5 μl 30% H_2_O_2_ and 10 ul luminol working solution. Negative controls contained 400 μl PBS and 10 μl luminol working solution. Chemiluminescence was measured for 15 min using a luminometer and ROS was reported as RLU/S/10^6^ sperm.

#### ROS by flow cytometer procedure

The intracellular ROS was determined according to WHO criteria [22] by DCFH-DA (25 μM) and DHE (1.25 μM) which were separately added to 1–3×10^6^ sperm/ml fractions and incubated at 25°C for 20 and 40 min, respectively, in the dark room. Each sample was analyzed using a flow cytometer with a 488 nm argon laser (Becton Dickinson FACScan, San Jose, CA, USA). Green fluorescence of DCFH-DA (500–530 nm) and red fluorescence of DHE (590–700 nm) were evaluated with excitation wavelength at 488 nm and emission wavelength at 525–625 nm in the FL-2 channel. PI was used as a counterstain dye for DCFH for the distinction of dead sperm. Data were expressed as the percentage of fluorescent spermatozoa.

#### Total antioxidant capacity

The specimens in each group were centrifuged at 300 g for 5 min and the cell-free seminal plasma was assessed for total antioxidant capacity using the TAC assay kit (catalog # JM-K274-100). Briefly, 100 μl of working solution (one part Cu^2+^ reagent with 49 parts of Assay diluent) and 100 μl of seminal plasma with 1μl protein mask were mixed in a centrifugation tube and incubate at room temperature for 1.5 hours. The absorbance of the formed colored complex was measured against the reagent blank at 570 nm using the plate reader.

#### Mitochondrial membrane potential

The stock solution of JC-1 (1.53 mM) was dissolved in DMSO. A 1mL aliquot of semen (3×10^6^ sperm/ml) was stained with 1.0 μl of JC-1 stock solution for 15 min at 37°C and centrifuged for 5 min at 800 g. The pellet was diluted 1:5 in PBS and immediately assessed for orange and green staining by flow cytometry.

For measurement of each sample, a total of 10,000 gated events based on the FS and SS were analyzed per sample using the flow cytometer. A 488 nm filter was used for excitation of JC-1 and emission filters of 530 and 575 nm were used to quantify the population of spermatozoa with green and orange fluorescence, respectively. Sperm with JC-1 staining was detected by FL1 (green) and FL2 (orange) canals.

#### Acrosome integrity

The acrosome integrity was evaluated using FITC-PSA staining method [22]. Briefly, specimen (10 μl) was smeared on the microscope slide, dried and fixed in ethanol at 20°C for 30 min. The smear was stained with FITC-PSA and incubated at 4°C for 60 min. The numbers of 300 sperm was evaluated in each replicate using fluorescence microscopy at 100× magnification at 450–490 nm excitation.

#### DNA fragmentation

The acridine orange test was performed as described by Chohan et al. [27] with slight modifications. A suspension of 30 μl of spermatozoa (10×10^6^ sperm/ml) was smeared onto a pre-cleaned glass slide, dried and fixed in Carnoy’s solution (1:3 ratio of methanol and glacial acetic acid) at 4°C for 10 min. Slides were air dried and stained with AO solution containing 0.15 mg/ml and purified AO in staining buffer (0.037 mol/L citric acid, 0.126 mol/L Na_2_HPO_4_.7H_2_O, 0.011 mol/L EDTA and 0.15 mol/L NaCl and pH adjusted to 6.0) for 5 min in the dark room. Slides were washed with PBS and the percentage of sperm with denaturated and double-stranded DNA was evaluated by counting at least 200 sperm using a fluorescent microscope in 100× magnification with an excitation wavelength of 450–490 nm. Sperm with intact double-stranded DNA was stained green and sperm with denatured DNA was stained red or orange fluorescence. DNA fragmentation index was calculated according to following equation:

$$\%DFI=\frac{Red\;fluorescence}{Total\;(red+green)fluorescence}\times 10^{2}$$ (3)

#### Externalization of phosphatidylserine (Annexin V/PI)

The PS flip-flop motion across the membrane was detected using the phosphatidylserine detection kit (IQ Products BV, Rozenberglaan 13a 9227 DL Groningen, the Netherlands). Briefly, samples were washed in 1 ml calcium buffer 1X and centrifuged at 800 g for 5 min. The pellet was re-suspended in 1 ml calcium buffer 1X and re-adjusted the cell concentration to 1 × 10^6^ sperm/ml. the 10 μl Annexin V FITC was added and incubated for 20 min at 4°C and the cells were rinsed again with calcium buffer. The 10 μl PI was then added to the cell suspension and incubated for at least 10 min at 4°C. Immediately, the stained sperm was analyzed using flow cytometry by measuring the fluorescence emission at 530 nm (FL1 canal) and 575 nm (FL3 canal). We designated the viable sperm as (An^-^/PI^-^), early apoptotic sperm as (An^+^/PI^-^), apoptotic sperm as (An^+^/PI^+^) and necrotic sperm as (An^-^/PI^+^).

### Statistical analysis

All experiments were analyzed using SPSS version 16 (SPSS Inc. Chicago, ILL). For the evaluation of physicochemical properties of water, Size (n = 300), tension (n = 300), viscosity (n = 300), and density (n = 300) were compared among the groups.

Data in physicochemical properties of water were analyzed by two-way analysis of variance (two-way ANOVA) and interactions between time periods and repetition rates for above-mentioned variables were considered as fixed factors in this model.

For the measurement of the data in the post-thaw sperm quality evaluations, the numbers of 25 normospermic semen samples were used and analysis was performed by one-way ANOVA. Afterward, a Tukey *post hoc* analysis was done for comparing between means.

P-values were adjusted by Bonferroni for multiple comparisons at 0.001 significantly level. For sperm parameters, P-values less than 0.05 were considered as significant.

## Results

### Physicochemical analysis of water

#### Cluster size measurement

**Fig 1:**
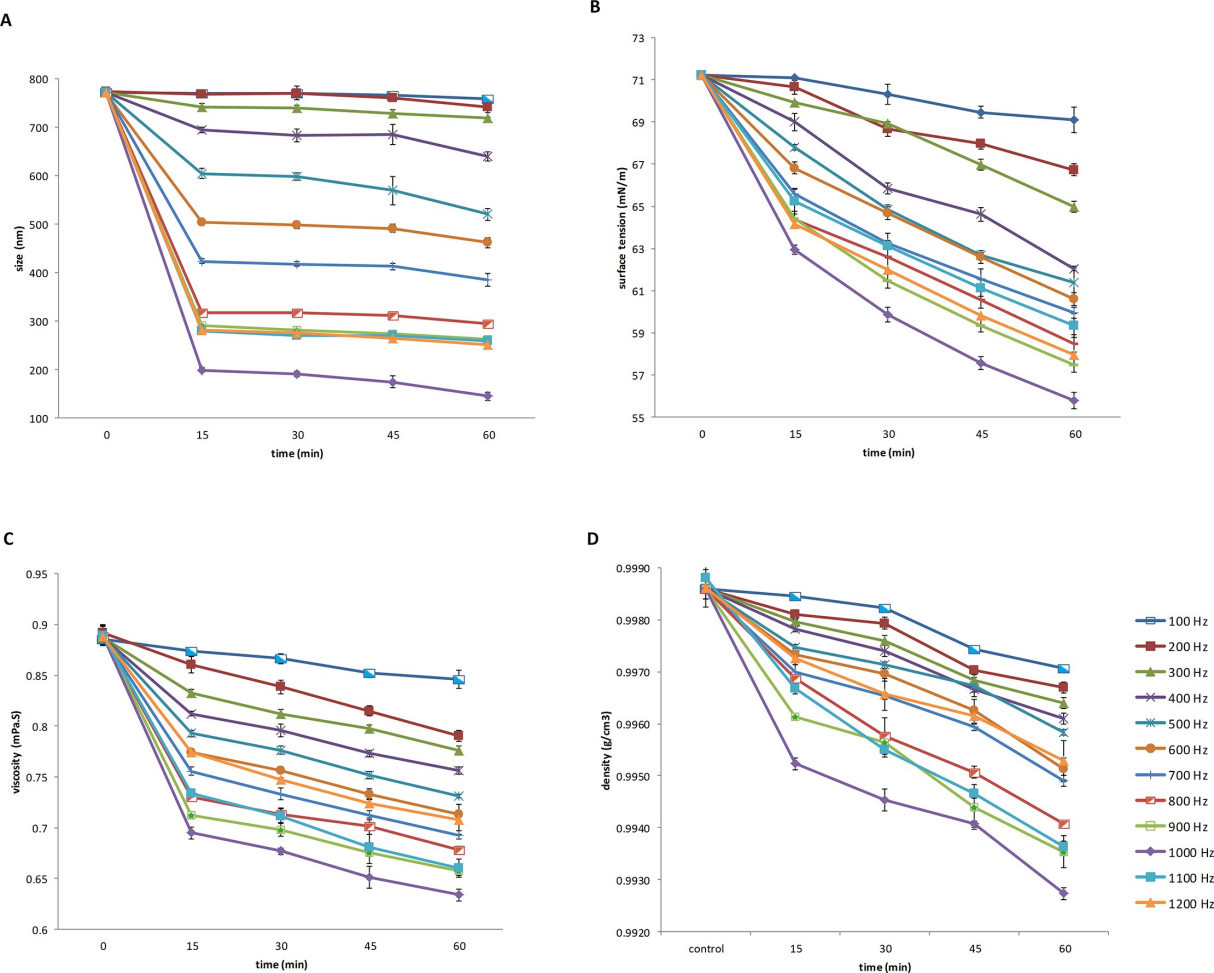
Physicochemical analysis of water. (A) Cluster size analysis. (B) Variation of the dynamic surface tension was measured by Tensiometer. (C) The viscosity of water was determined using a rheometer. (D) The density of water was assessed by a densitometer.

Cluster size was significantly affected by the repetition rate and time period (P < 0.001; S1A Table). The average of cluster size was significantly decreased with increasing repetition rate and time periods. As shown in Fig 1A, the highest cluster size was observed in 100 Hz repetition rate with 15 min time period and the lowest cluster size was found in 1000 Hz repetition rate with 60 min time period. Furthermore, as shown in S2A and S3A Tables, the size of water clusters (with exception of 100 and 200 Hz repetition rates) following the treatment with ELEFs was significantly changed (P<0.001). Pairwise comparisons of repetition rates showed significant differences among groups (S3A Table).

#### Surface tension and viscosity of water measurements

The effects of time periods and repetition rates showed significant differences in surface tension and viscosity characteristics (P < 0.001; S1B and S1C Table). Fig 1B shows that the dynamic surface tension of water was reduced accompanied by an increase in repetition rates and time periods. The highest surface tension was found in 100 Hz repetition rate with 15 min time period and the lowest surface tension was observed in 1000 Hz repetition rate with 60 min time period. As shown in S2B and S3B Tables, except for 200 vs. 300, 300 vs. 400, and 400 vs. 500 Hz repetition rates, the differences in water surface tension were significant (P < 0.001).

Also, according to Fig 1C, the viscosity of water was significantly decreased as a result of increasing time periods and repetition rates. Differences in viscosity for all time periods and repetition rates test were significant (P < 0.001; S1C and S2C Tables).

#### Density measurement

Statistical analysis of data demonstrated significant differences between repetition rate and time periods for water density (P < 0.001; S1D Table). The water density determination results are presented in Fig 1D. Water density was reduced as the time period and repetition rate were increased. The highest density was observed in 100 Hz repetition rate with 15 min and the lowest density was found in 1000 Hz repetition rate with 60 min. Pairwise comparisons between repetition rate and time period effects on physicochemical characteristics were significant, respectively (P < 0.001). Pairwise comparisons in 200 vs. 100, 200 vs. 300, 500 vs. 600, 500 vs. 700, and 600 vs. 700 Hz repetition rates did not show any significant difference. The effects of times periods were significant (P < 0.001; S2D and S3D Tables). Pairwise comparisons between repetition rate and time period effects on physicochemical characteristics were significant, respectively (P < 0.001). Pairwise comparisons in 200 vs. 100, 200 vs. 300, 500 vs. 600, 500 vs. 700, and 600 vs. 700 Hz repetition rates did not show significant differences. The effects of times periods were also significant (P < 0.001; S2D and S3D Tables).

#### Water memory

**Fig 2:**
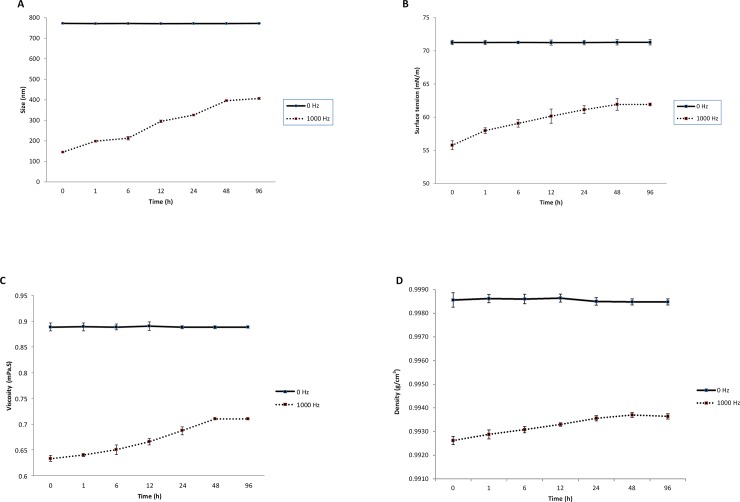
The memory effect of electromagnetized water. (A) Cluster size analysis. (B) Variation of the dynamic surface tension. (C) The viscosity of water. (D) The density of water. In all sub-graphs the physicochemical properties of water do not return to the point before applying 1000 Hz ELEF. Values are expressed as mean ± SD; n = 5. P-values were adjusted by Bonferroni for multiple comparisons at 0.001 significantly level.

Fig 2 shows that the average of cluster size, surface tension, viscosity, and density of the network of water molecules following the treatment with 1000 Hz ELEF were significantly (P < 0.001) increased gradually with increasing time, but finally, they do not return to the point before applying ELEF (it shows as 0 HZ in Fig 2), and the physicochemical properties become stable at a certain value at times of 48 and 96.

### Post thaw evaluation of sperm

#### Motion characteristics

Post-thaw sperm parameters after the cryopreservation in fresh control, frozen control, and frozen ELEF are presented as mean ± SD in Table 1. Total motility and progressive parameters were significantly higher in fresh control (P < 0.05) than in frozen control and frozen ELEF. Total and progressive motility were significantly (P < 0.05) higher in frozen ELEF than in frozen control. Moreover, significantly (P < 0.05) lower numbers of immotile sperm was observed in fresh control and frozen ELEF as compared to frozen control. This parameter was significantly (P < 0.05) lower in frozen ELEF than in fresh control.

**Table 1:** Effects of freezing medium prepare from treated and untreated waters on sperm motility. The assigned letters of a, b and c indicate significant differences (p < 0.05) among the groups. Values are expressed as mean ± SD, Tukey test; n = 25. P-value adjustment for multiple comparisons: Bonferroni.

Motility (%)	Fresh Control	Frozen Control	Frozen ELEF
**Total motility**	74.03 ± 10.53^a^	31.35 ± 3.49^c^	49.52 ± 5.26^b^
**Progressive**	53.85 ± 13.34^a^	12.11 ± 4.41^c^	27.98 ± 7.88^b^
**Non-progressive**	20.06 ± 7.36	19.24 ± 3.68	21.54 ± 6.62
**Immotile**	25.96 ± 10.52^c^	68.64 ± 3.49^a^	50.47 ± 5.26 ^b^

The variables of VCL, VSL, VAP, LIN, STR, ALH and BCF were significantly higher in fresh control (P < 0.05) than in frozen control and frozen ELEF. In addition, Frozen ELEF produced the higher VCL, VSL, and VAP as compared to frozen control. The LIN, STR, ALH, and BCF were not significant differences between frozen groups (Table 2).

**Table 2:** Effects of freezing medium on sperm motion variables in frozen control and frozen ELEF. The assigned letters of a, b and c indicate significant differences (p < 0.05) among the groups. Values are expressed as mean ± SD, Tukey test; n = 25. P-value adjustment for multiple comparisons: Bonferroni.

Motion Variables	Fresh Control	Frozen Control	Frozen ELEF
**VCL (μm/s)**	86.43 ± 21.09^a^	41.89 ± 11.87^c^	55.16 ± 18.19^b^
**VSL (μm/s)**	41.52 ± 13.54^a^	14.91 ± 8.35^c^	23.47 ± 11.42^b^
**VAP (μm/s)**	55.17 ± 14.65^a^	22.1 ± 8.81^c^	30.6 ± 12.85^b^
**LIN (%)**	48.66 ± 7.48^a^	34.67 ± 13.35^b^	36.56 ± 15.05^b^
**STR (%)**	75.44 ± 7.91^a^	63.68 ± 15.81^b^	65.41 ± 16.21^b^
**ALH (μM)**	2.26 ± 0.47^a^	1.52 ± 0.53^b^	1.84 ± 0.67^b^
**BCF (Hz)**	16.54 ± 3.87^a^	10.59 ± 6.22^b^	11.13 ± 5.72^b^

#### Viability

**Fig 3:**
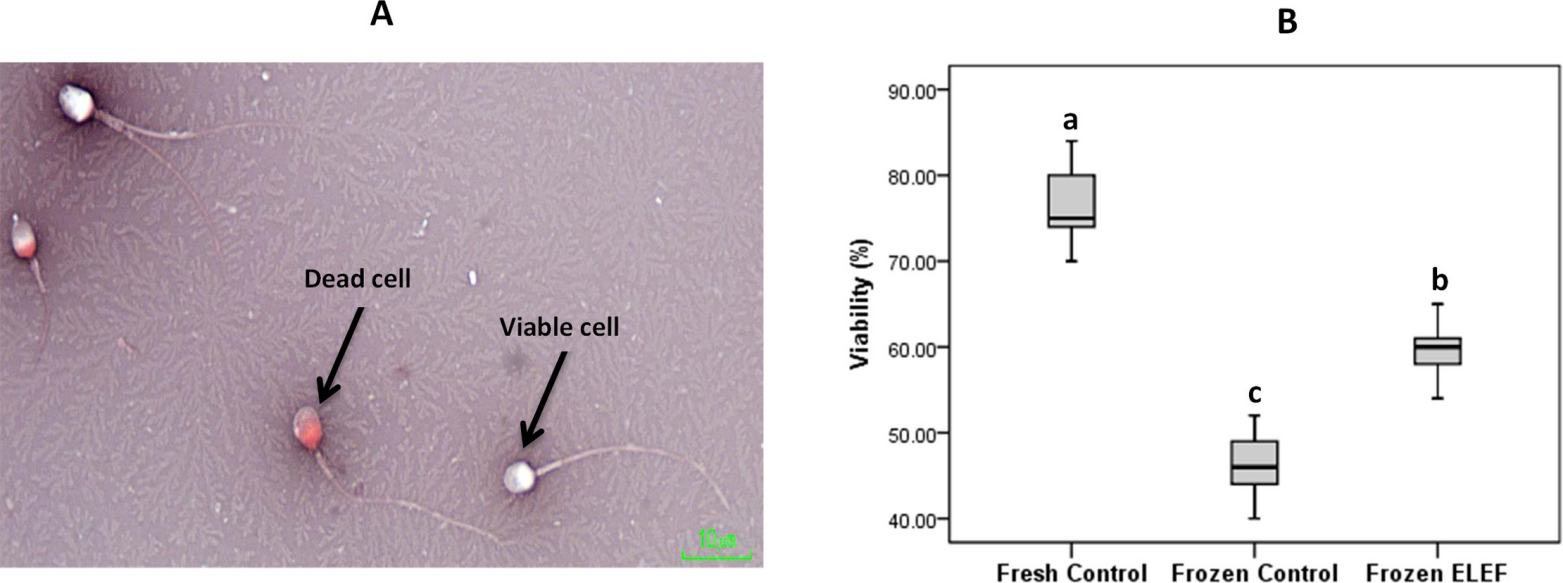
Effect of ELEF on percentages of living sperm. (A) Photography of dead (red) and viable (white) spermatozoa in eosin-nigrosin staining. (B) Box-and-whisker plots for sperm viability. The boxes represent the 25th and 75th percentiles; whiskers are lines extending from each end of the boxes covering the extent of the data on 1.5× inter-quartile range. Middle lines that bisect the boxes represent the median value. The percentage of viable sperm in frozen control was significantly lower than that in fresh control and frozen ELEF (at 1000 Hz repetition rate with 60 min time period) using eosin-nigrosin protocol. The assigned letters of a, b and c indicate significant differences (p < 0.05) among the groups. Values are expressed as mean ± SD.

Table 3 and Fig 3 show the percentage of live sperm in different experimental groups. A significantly (P < 0.05) higher percentage of viability was observed in frozen ELEF in comparison with frozen control. This value was significantly lower in both freezing groups as compared to fresh group (P < 0.05).

**Table 3:** Cellular parameters of sperm investigated between three experimental groups. The assigned letters of a, b and c indicate significant differences (p < 0.05) among the groups. Values are expressed as mean ± SD, Tukey test; n = 25. P-value Adjustment for multiple comparisons: Bonferroni.

Parameter	Fresh Control	Frozen Control	Frozen ELEF
**MDA levels in semen (nmol/ml)**	31.31 ± 1.95^b^	39.62 ± 2.47^a^	32.78 ± 2.26^b^
**MDA levels in sperm (μM/20×10**^**6**^**sperm)**	3.36 ± 0.51^b^	9.98 ± 0.58^a^	3.67 ± 0.61^b^
**Seminal TAC (nmol/μl)**	19.52 ± 0.75^a^	8.93 ± 0.66^c^	15.50 ± 0.68^b^
**Seminal ROS (RLU/S/10**^**6**^**sperm)**	26.63 ± 1.19^c^	55.387± 3.01^a^	41.93 ± 1.65^b^
**Viability (%)**	76.28 ± 4.00^a^	46.44 ± 3.40^c^	59.28 ± 2.76^b^
**Abnormal Morphology (%)**	81.76 ± 1.98^c^	96.52 ± 3.12^a^	88.04 ± 4.29^b^
**Intact Membrane (%)**	88.24 ± 1.71^a^	36.76 ± 2.86^c^	64.40 ± 4.93^b^
**DCFH-DA Level (%)**	7.88 ± 1.09^c^	32.72 ± 2.56^a^	20.52 ± 2.02^b^
**DHE Level (%)**	6 ± 0.82^c^	31.64 ± 1.98^a^	15.84 ± 1.99^b^
**JC-1 Staining (red/green ratio)**	0.96 ± 0.04^a^	0.27 ± 0.05^c^	0.66 ± 0.07^b^
**Acrosome Intact (%)**	94.64 ± 3.44^a^	57.88 ± 5.13^c^	77.84 ± 4.12^b^
**DNA Fragmentation Index (%)**	6.52 ± 1.23^c^	17.76 ± 2.98^a^	10.44 ± 1.00^b^

#### Morphology and membrane integrity

**Fig 4:**
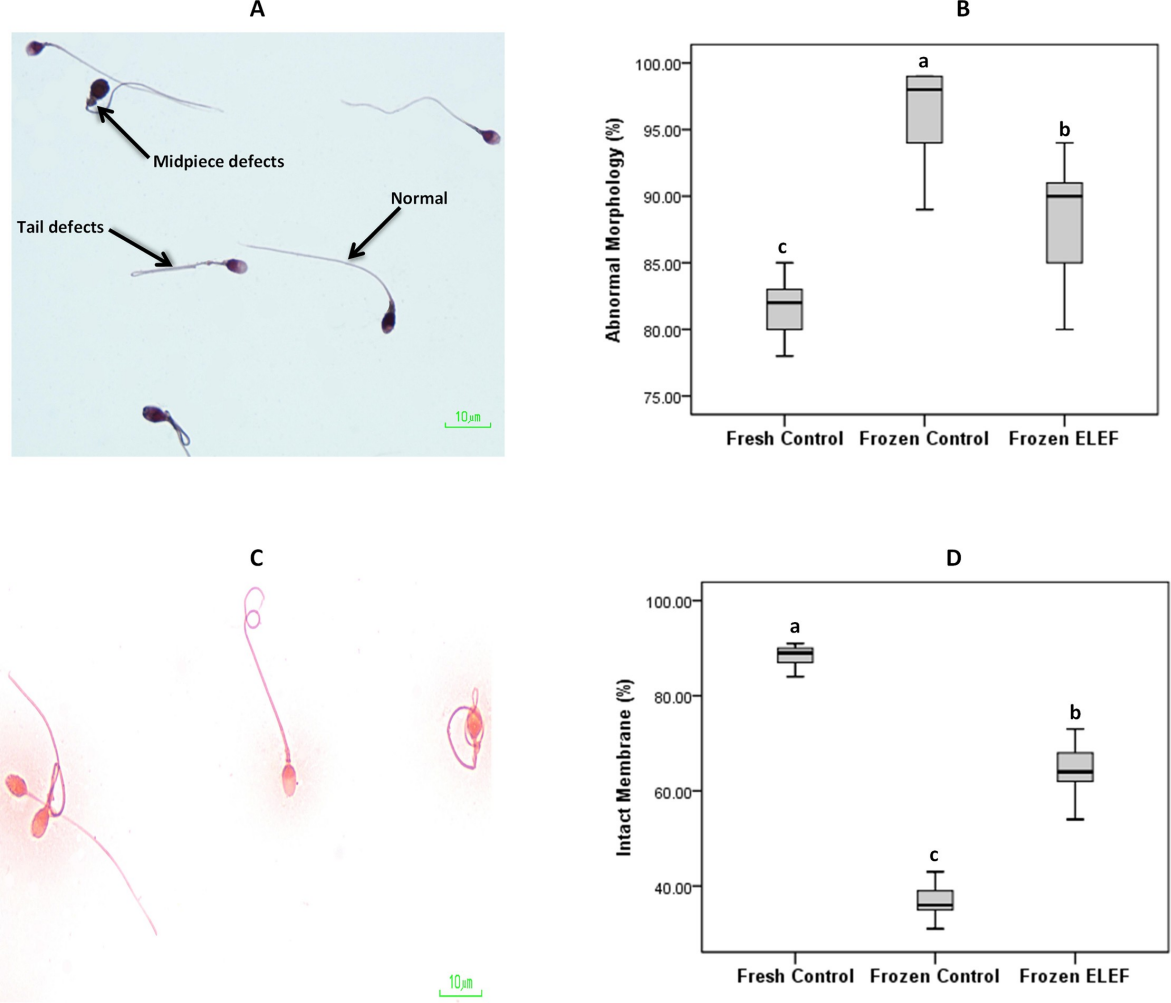
Morphology and membrane integrity. (A) Sperm morphology test; the various types of sperm head, neck and tail defects are analyzed. (B) Box-and-whisker plots for sperm with abnormal morphology from the three groups. Abnormal morphology significantly differs between experimental groups. (C) Graph of sperm exhibiting tail swelling under hypo-osmotic solution as indicated by tail curling while dead sperm show no change. (D) Box-and-whisker plots for sperm with intact membrane from the three experimental groups. Percentage of intact membrane is lower in frozen control than in fresh and frozen ELEF. The assigned letters of a, b and c indicate significant differences (p < 0.05) among the groups. Values are expressed as mean ± SD.

The mean of sperm abnormal morphology was significantly lower in fresh control sperm as compared to frozen control and frozen ELEF (Table 3, Fig 4A and 4B). The difference for this value between frozen control and frozen ELEF was also significant (P < 0.05). The HOST results showed that percentage of intact plasma membrane was significantly (P < 0.05) higher in fresh control as compared to frozen control and frozen ELEF. This value was higher significantly (P < 0.05) in frozen ELEF than in frozen control (Table 3, Fig 4C and 4D).

#### Lipid peroxidation

The MDA level in seminal plasma and sperm were significantly (P < 0.05) lower in frozen ELEF than frozen control. This value was not significantly (P < 0.05) different between fresh control and frozen ELEF (Table 3).

#### ROS by chemiluminescence and flow cytometry

As shown in Table 3, the ROS levels in the frozen control were significantly (P < 0.05) higher than fresh control and frozen ELEF.

**Fig 5:**
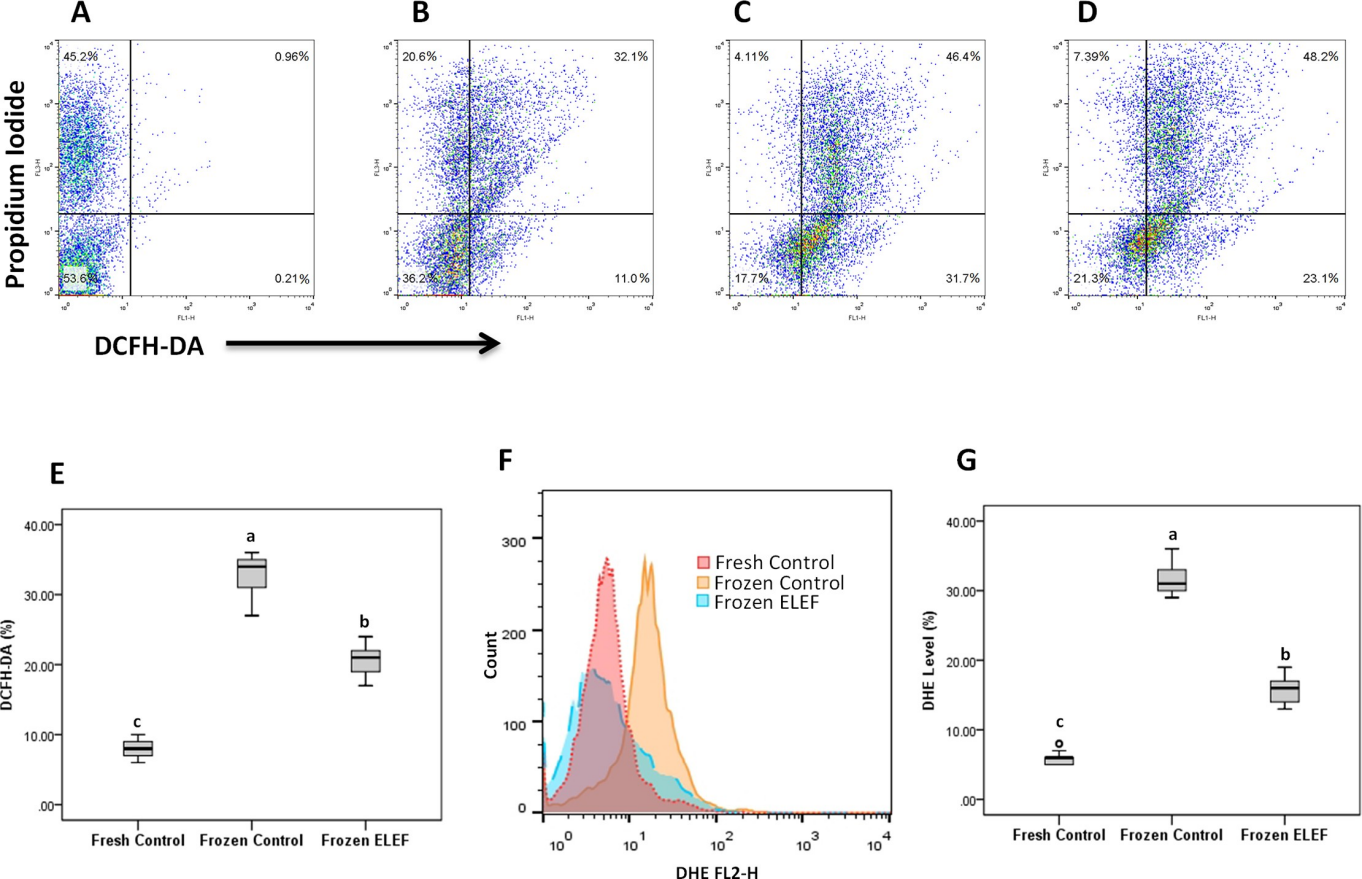
ROS in three group specimens were analyzed using flow cytometry by staining with PI/ DCFH-DA-FITC and DHE. Flow cytometry of specimens stained with PI/ DCFH-DA (A-E) and DHE (F and G). Panel A represents PI control without DCFH-DA, panels B-E represent ROS production in the live or dead sperm population. Panel F and G represent DHE levels in fresh and frozen controls and frozen ELEF. The assigned letters of a, b and c indicate significant differences (p < 0.05) among the groups. Values are expressed as mean ± SD.

Table 3 and Fig 5 show the ROS levels assessed by flow cytometry in populations of the live and dead sperm in three groups. The higher significant positive signal of DCFH was observed in frozen control than those obtained in fresh control and frozen ELEF. The percentage of DHE in the frozen control was significantly increased as compared to fresh control and frozen ELEF (P < 0.05). These values were significantly higher (P < 0.05) in frozen ELEF as compared to fresh control.

#### Total antioxidant capacity

Table 3 shows the effect of ELEF cryopreservation on the total antioxidant capacity of human semen as evaluated by TAC assay kit. There was a significant reduction (P < 0.05) in the TAC concentration in frozen control rather than fresh control and frozen ELEF. This value was significantly (P < 0.05) lower in frozen ELEF as compared to fresh control.

#### Assessment of mitochondrial membrane potential

**Fig 6:**
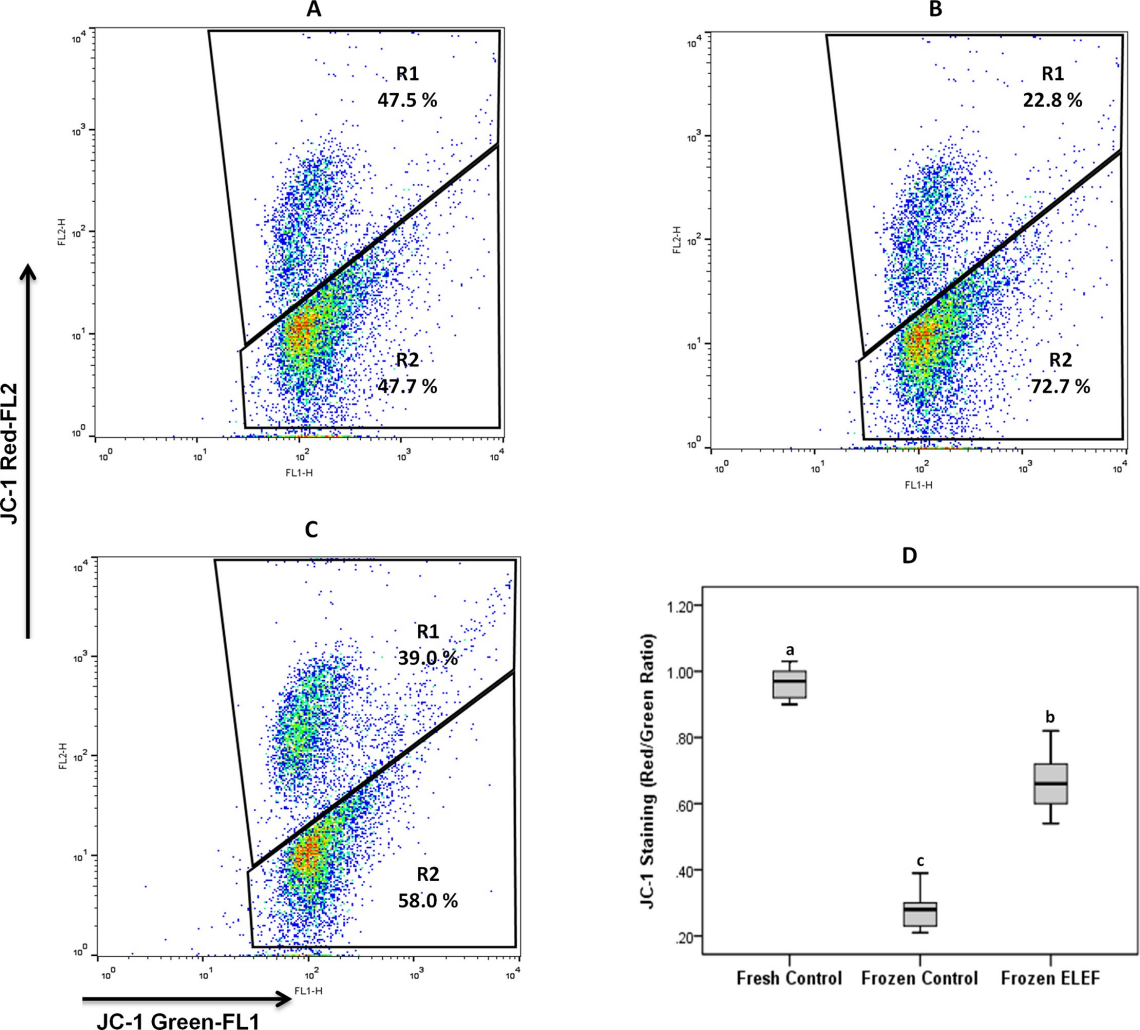
**Representative flow cytometry for (A) fresh control stained with JC-1; (B) frozen control sperm stained with JC-1; (C) frozen ELEF. Box-and-whisker plots for sperm MMP from the three experimental groups (D).** The percentage values represent the proportion of sperm with high MMP (top right quadrant) and low MMP (bottom right quadrant). The assigned letters of a, b and c indicate significant differences (p < 0.05) among the groups. Values are expressed as mean ± SD.

Table 3 and Fig 6 show a sharp shift in the red fluorescence to green in different experimental groups. The ratio of red/green fluorescence in frozen control was significantly reduced (P < 0.05) as compared to fresh and frozen ELEF. This ratio was significantly higher in frozen ELEF than fresh control (P < 0.05).

#### Acrosome integrity

**Fig 7:**
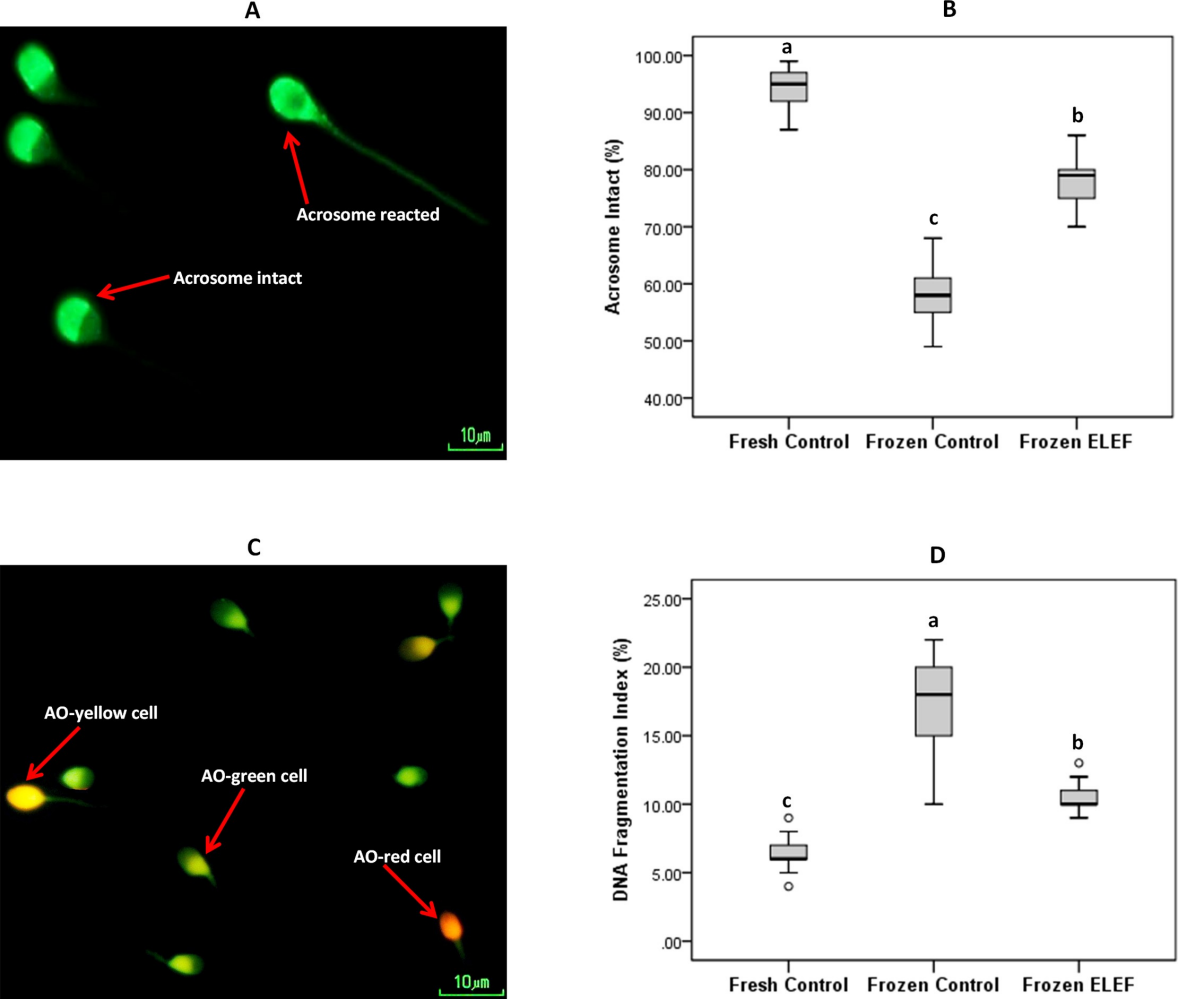
Acrosome integrity and sperm DNA fragmentation. (A) Acrosome evaluation was detected using FITC-PSA staining; acrosome intact and acrosome-reacted are shown. (B) Box-and-whisker plots for sperm with acrosome intact from the three experimental groups show the significant differences among all groups (C) Acridine orange test in human sperm; sperm heads with double-stranded DNA were green (AO-green cells) and sperm heads with single-stranded DNA were red or yellow (AO-red or AO-yellow cells). (D) Box-and-whisker plots for DNA fragmentation index of sperm from the three experimental groups. The higher DNA fragmentation index is observed in frozen control as compared to fresh control and frozen ELEF. The assigned letters of a, b and c indicate significant differences (p < 0.05) among the groups. Values are expressed as mean ± SD.

The acrosome integrity of sperm detected by FITC/PSA stain is presented in Table 3, and Fig 7A and 7B. The acrosome integrity was significantly higher in the frozen ELEF than frozen control (P < 0.05). Also, this index was significantly higher in fresh control as compared to other groups (P < 0.05).

#### Sperm DNA fragmentation

Specimen stained with acridine orange showed that the DNA fragmentation index was significantly reduced (P < 0.05) in fresh and frozen ELEF in comparison with frozen control. In addition, this value was significantly (P < 0.05) different between fresh control and frozen ELEF (Table 3, Fig 7C and 7D).

#### Annexin V/Propidium iodide apoptosis assay

**Fig 8:**
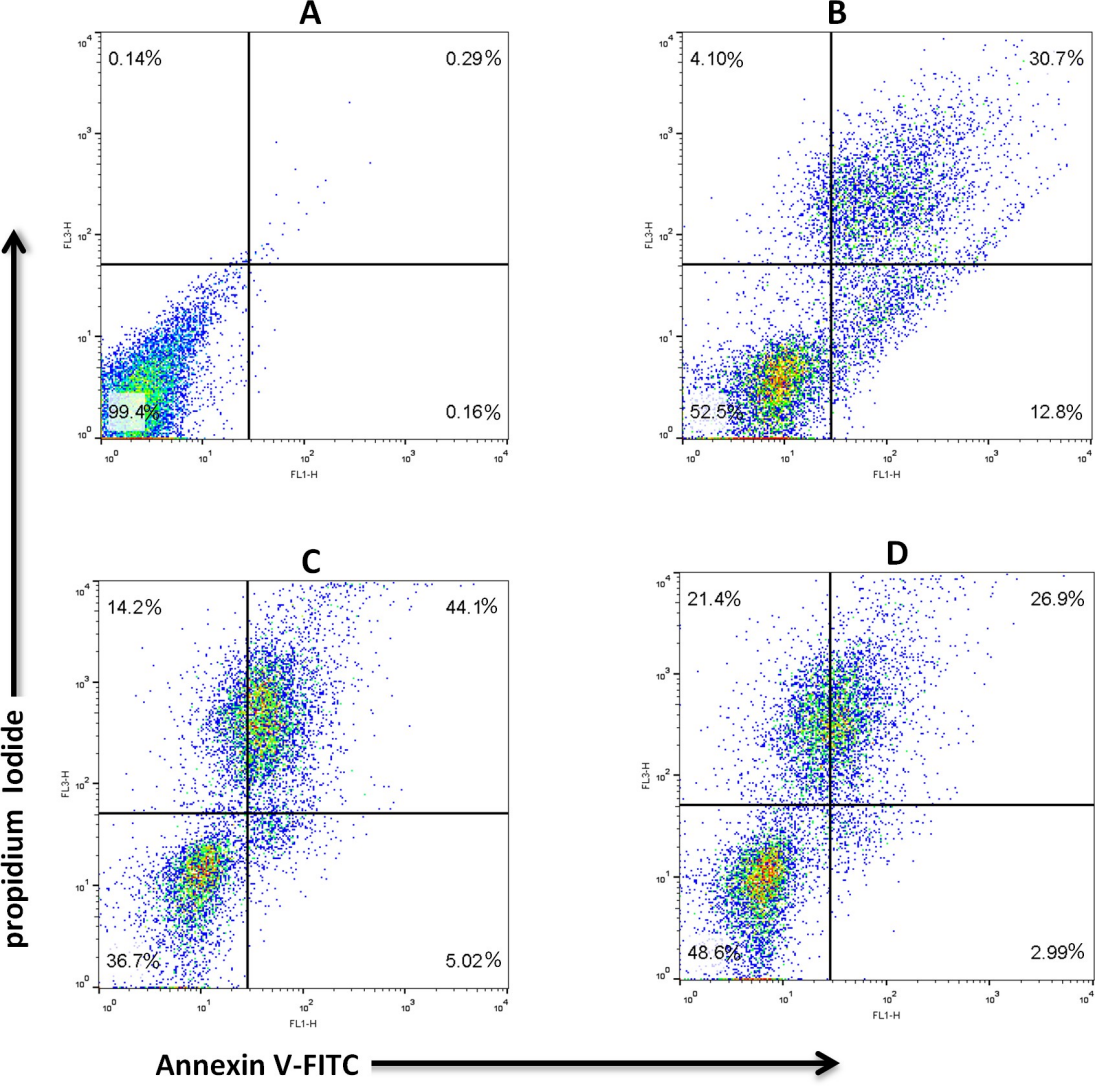
Annexin V and propidium iodide staining were used to determine the different cell populations. (A) Unstained sperm. (B) A fresh control. (C) A frozen control. (D) Frozen ELEF. The top left quadrant represents necrotic cells. The lower left quadrant shows live cells. The lower right quadrant and the top right quadrant show early apoptotic and late apoptotic cells, respectively.

**Fig 9:**
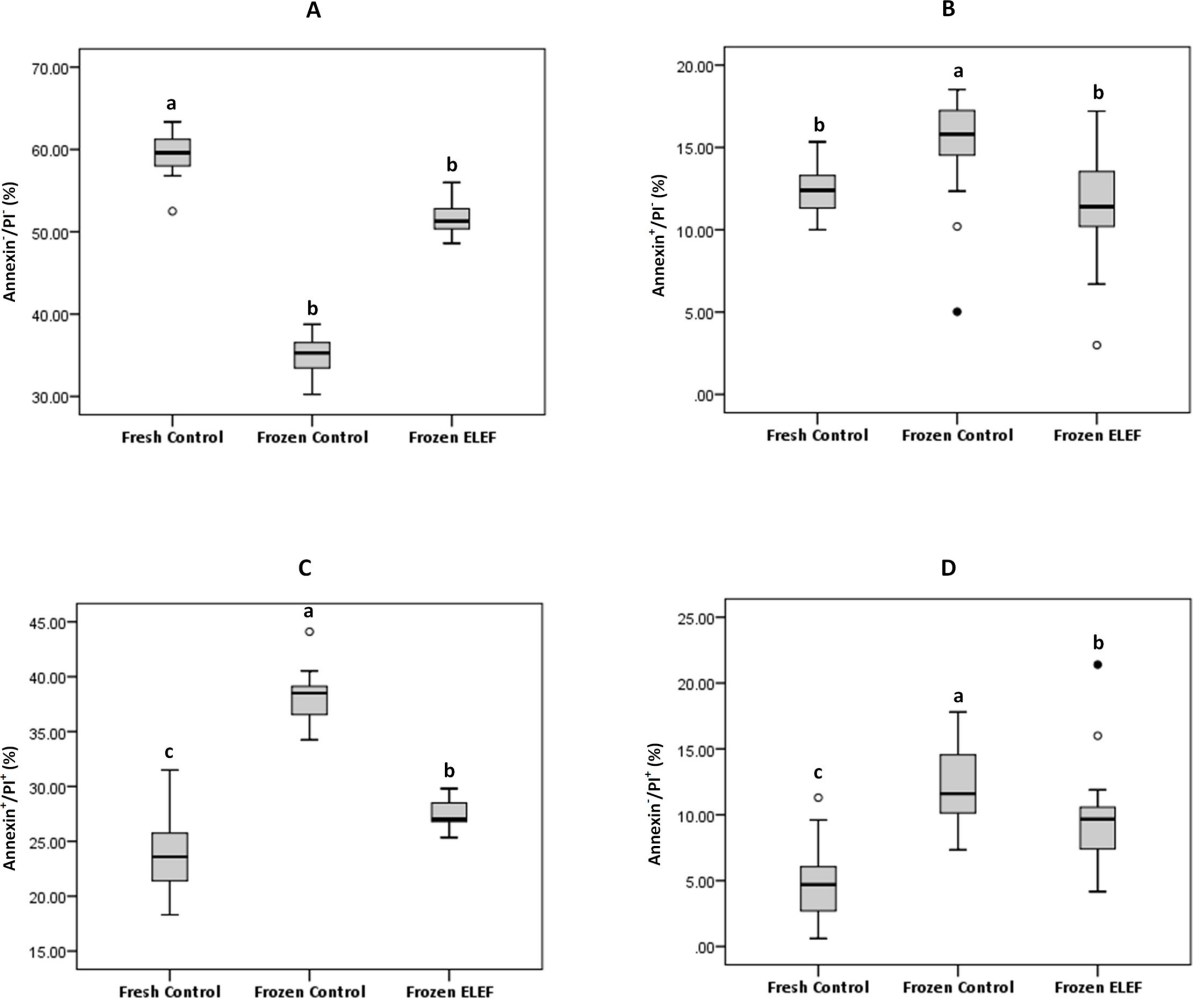
**Box-and-whisker plots for (A) Annexin V**^**-**^**/PI**^**-**^**, (B) Annexin V**^**+**^**/PI**^**-**^**, (C) Annexin V**^**+**^**/PI**^**+**^**, and (D) Annexin V**^**-**^**/PI**^**+**^ **staining of sperm to determine the different cell populations.** The box and whisker plots show the median to interquartile ranges. Lower and upper whiskers represent 5th and 95th percentiles, respectively. The boxes represent the 25th and 75th percentiles. The assigned letters of a, b and c indicate significant differences (p < 0.05) among the groups. Values are expressed as mean ± SD.

Labeling of sperm with PI and Annexin V allows identifying four populations of sperm (Table 4, and Figs 8 and 9). The proportion of necrotic spermatozoa (An^-^/PI^+^) in frozen control was significantly (P < 0.05) higher than fresh control and frozen ELEF. The proportion of apoptotic spermatozoa with An^+^ and PI^+^ was significantly (P < 0.05) lowered in fresh control and frozen ELEF with respect to frozen control. The populations of early apoptotic sperm (An^+^/PI^-^) were significantly (P < 0.05) lower in frozen ELEF and fresh control with regard to frozen control, while there were no significant differences between fresh and frozen ELEF groups. The viable sperm (An^-^/PI^-^) was significantly (P < 0.05) higher in fresh control and frozen ELEF when compared to frozen control.

**Table 4:** Staining of sperm by Annexin V and propidium iodide (PI). The assigned letters of a, b and c indicate significant differences (p < 0.05) among the groups. Values are expressed as mean ± SD, Tukey test. An^-^/PI^-^, live cells; An^+^/PI^-^, early apoptotic cells; An^+^/PI^+^, late apoptotic cells; An^-^/PI^+^, necrotic cells.

Sample	N	An^-^/PI^-^ (%)	An^+^/PI^-^ (%)	An^+^/PI^+^ (%)	An^-^/PI^+^ (%)
**Fresh Control**	25	59.51 ± 2.33^a^	12.27 ± 1.43^b^	23.58 ± 3.45^c^	4.62 ± 2.79^c^
**Frozen Control**	25	35.03 ± 2.23^c^	15.32 ± 3.01^a^	38.11 ± 2.07^a^	12.00 ± 2.81^a^
**Frozen ELEF**	25	51.65 ± 1.65^b^	11.47 ± 2.93^b^	27.27 ± 1.23^b^	9.58 ± 3.58^b^

## Discussion

Many studies have demonstrated that magnetic fields affect the physicochemical properties of water [28–32], but a few studies have examined the impact of electromagnetic fields on the physicochemical characteristics of liquid water. Also, these studies investigated the ice crystal properties and they did not evaluate the properties of liquid water [20, 33–36]. In our experiment, we evaluated whether electromagnetic field-treated water can be used for the preparation of freezing media and the cryopreservation of human sperm.

We assumed that ELEFs may change the physicochemical properties of water and reduce the ice crystal formation in cryopreservation media during the sperm freezing process that would lead to better preservation of sperm in this context. We found that only one study investigated the effect of employing magnetized medium for the cryopreservation of boar sperm and evaluated the membrane damage and fertilizing potential after thawing [37]. However, there is no available evaluation corresponding to the physicochemical properties of the magnetized medium.

In the present study, all mentioned gaps were filled and a comprehensive study was conducted to evaluate the physicochemical properties of water to assess the possibility of using ELEFs for preparation of human sperm freezing media. It has been reported that water has not a monotonic structure [30, 38]; therefore, water molecules can produce the assemblies (such as clusters). However, this behavior could be changed by the external factors [39]. In the first series of tests, we measured the size of created water clusters. As shown in Fig 1, different sizes of the cluster (nm) were observed in ELEFs-treated water showing the presence of different clusters and domains. As previously shown, the main force in water models and structures stems from the presence of hydrogen bonds, and physicochemical properties of water are mostly attributed to this feature [39]. There are two separate areas of water including cluster and bulk water that can be distinguished by hydrogen bond networks [39]. When water molecules enter a cluster, it becomes larger and subsequently, the hydrogen bonds will become shorter and stronger. Also, the cluster structure becomes smaller when the water molecules leave the cluster; the molecules become far from each other in these clusters and hydrogen bonds become longer and hence weaker [40, 41]. In the present study, after applying ELEFs for water, the size of the clusters was different in the range of 144–742 nm. We chose the lowest size of the cluster (144 nm) to prepare the cryopreservation media using the water-treated media exposed to the repetition rate of 1000 Hz with 60 min time period (Fig 1A).

The second series of assessments were done to measure the surface tension significantly reduced by increasing repetition rates and time periods (Fig 1B). Where the surface tension or the Gibbs free energy per unit area is reduced, it can be concluded that the hydrogen bonds between the clusters are broken and less force is required to break the surface. It seems that this sharp reduction in surface tension in 1000 Hz ELEF- treated water is due to the dilution of coherent domains and the ratio of the cluster of the water molecule to bulk water led to an increase in the distances among the molecules in the cluster. It also results in smaller clusters of water as lower numbers of molecules participate in the structure of a cluster.

The water density was another important parameter in which was reduced in water treated with 1000 Hz ELEF. It has been reported that electromagnetic waves can increase the water temperature due to fluctuations and disturbances induced by ELEFs [42, 43]. As the temperature increases, the kinetic energy of the molecules increases and the higher vibration is induced. Therefore, the density will be reduced as the molecules demand more space [44]. Moreover, the higher temperature of the water treated with ELEFs can also reduce the viscosity of the water observed in the present study. This reduction is accompanied by the decrease in the clusters size with the regular structure.

The values for the viscosity of water obtained in this project (Fig 1D) are in agreement with those obtained by previous studies [45, 46]. In the present study, physicochemical characteristics of water (surface tension, density, viscosity, and cluster size) were optimally achieved at the repetition rate of 1000 Hz with 60 min time period.

We were also concerned about the surface tension, viscosity, density, and especially the clusters size of the network of water molecules after applying ELEFs could increase or go back to normality over time. Therefore, we conducted a water memory test for the physicochemical changes. We empirically found that the effect of electromagnetized water not only did not vanish, but also remained for a very long time. This suggests that if physical behavior induced into water molecules, this alteration remains protected in the memory of water. Our results are in line with previous studies on water memory who stated that creation of a physicochemical changes in the molecular network of water remains stable for a long time [47–49].

In our study, we evaluated the post-thaw quality of sperm after the cryopreservation in medium prepared with the water treated with 1000 Hz ELEF. We observed an improvement in many indicators of sperm functions including the motility and velocity parameters, viability, membrane functionality, apoptotic status, mitochondria function, acrosome integrity, DNA integrity, ROS level, and total antioxidant capacity of sperm after thawing in frozen ELEF.

The higher quality of the cryopreserved sperm in freezing media treated with 1000 Hz ELEF seems to be related to the small size of water clusters that in turn leads to the formation of smaller ice crystals and an increase in sperm survival during the frozen-thawed process. Also, improvement of post-thawing sperm in this study was probably due to the reduction of ice nucleation of crystal in freezing media treated with ELEF. It has been suggested that ELEF inhibit the ice nucleation at higher degrees of supercooling inducing delays in the initiation of ice nucleation [20].

Furthermore, it has been proposed that ELEF can increase the temperature of the freezing media by increasing the speed of the thawing process which consequently, reduced the probability of re-crystallization of the ice around of the sperm leading to less cryo-injury to the sperm membrane. Our findings in this parts are in agreement with Lee et al. 2015 who reported that magnetized extender improves the viability and fertilizing ability of boar sperm during a long-term liquid storage [37, 50]. However, there is much less known about the beneficial effects of EMFs exposure to the reproductive performance and the semen quality [51–57]. Other reports also revealed that the direct exposure of sperm to magnetic or electromagnetic fields may have the detrimental effects on the sperm [52, 58–61]. It seems that the direct exposure to either EMF or ELEF damages the sperm. For this reason, we exposed the freezing media to ELEF before adding the sperm.

One of the important parameters assessed in our study was the ROS concentration that was evaluated in two forms of extracellular and intracellular forms in thawed sperm. We found the ELEF reduced this index in both sperm and semen. It has been reported that ROS production during the cryopreservation is considered the main damage occurred in the structural and biochemical organelles of sperm [62]. This finding is in agreement with Sang et al. who reported that magnetic field reduces the ROS concentration in boar semen [37]. It has been also suggested that water subjected to electrolysis has a lower oxidation-reduction potential that can act against the ROS production [63]. We also found that data related to sperm quality stem from the ROS changes that are in agreement with Emamverdi et al. suggesting ROS is the main causation of lower quality sperm parameters during the cryopreservation [64]. Therefore, the higher motility, membrane functionality and viability of thawed sperm in frozen ELEF in the present study could be probably attributed to the lower concentration of ROS.

We also observed a reduction in total antioxidant capacity in both frozen groups in comparison with fresh. Such reduction was predictable because the freezing process alters the NADPH oxidase in the plasma membrane and subsequently induces the alterations in the electron transfer chain of the mitochondria leading to the generation of ROS [65, 66] considered the main culprit of reducing the total antioxidant capacity in sperm and semen. It seems that the alteration in electron transfer chain of the mitochondria was less in the frozen ELEF due to the lower production of ROS during the cryopreservation.

Mitochondrial membrane potential (MMP) was another parameter of thawed sperm significantly improved in frozen ELEF. Mitochondrion has a crucial function in sperm because the oxidative phosphorylation and ATP production occur in this organelle. It has been proposed that ROS reduce the NADH pool ($\frac{NADH}{{NAD}^{+}}$) resulting in electron transfer chain deficiency and therefore the ATP depletion [67, 68]. Therefore, the higher MMP in the frozen ELEF seen in this study might be linked to the reduction of ROS concentration providing a better protection in this pathway.

We assessed the acrosome integrity in this study because acrosome of sperm is changed during the cryopreservation. Due to the alteration in calcium channel and plasma membrane during cryopreservation, the damage to acrosome is probably [69]. The positive effects of ELEF were observed in the acrosome integrity of thawed sperm that is inconsistent with the finding of Lee et al. [37] who stated that magnetic field can protect the acrosome against ROS. Moreover, the DNA integrity of sperm a strong biomarker for fertilizing ability had a similar trend with the acrosome integrity in frozen ELEF. It seems that ROS is the key reason that has detrimental effects on both acrosome and the DNA stability.

It has been stated that the excess concentration of ROS leads to produce of 8-Oxo-2'-deoxyguanosine that is one of the major oxidized products during the DNA oxidation giving rise to single DNA strand breaking [70]. Therefore, another reason for the lower percentage of DFI in frozen ELEF could be pertained to the lower production of 8-Oxo-2'-deoxyguanosine as result of the ROS reduction.

Finally, we identified four populations of sperm via probing them with Annexin V and PI staining in our experimental groups. The fraction of An^-^ and PI^+^ sperm is probably either necrotic sperm with the high degree of membrane disorganization which cannot bind to Annexin V or sperm in the later stage of apoptosis [71]. We observed that the population of viable sperm (An^-^/PI^-^) was reduced in both freezing groups as compared to fresh state. This reduction is in parallel with the findings of earlier studies [72, 73]. Ramos et al. [74] claimed that over-oxidation and the low total number of thiol groups during freezing process induce apoptosis in sperm. We also found that reducing the population of viable sperm is much less in the frozen control as compared to frozen ELEF. This phenomenon can be associated with the lower concentration of ROS in the frozen ELEF.

In our study, we observed a logical relationship among ROS, mitochondria active potential, apoptosis, and the DNA damage that were all improved in the frozen ELEF group. It would be plausible that ROS-induced DNA damage probably induces the apoptotic pathway in sperm because there are strong correlations among apoptosis, DNA damage and ROS levels [75]. Furthermore, ROS oxidized the mitochondrial pores leading to the release of cytochrome c due to disruption of the mitochondrial membrane potential (MMP) [76]. Cytochrome c also induces the activation of initiator caspases using the aggregation of adapter proteins to Apaf-1 formation and initiation of apoptosis [77, 78].

## Conclusion

Several quality indicators of human sperm after freeze-thaw improved in a freezing medium prepared with the exposed water to 1000 Hz ELEF. It can be considered as useful strategy for increasing the fertilizing potential of thawed semen. This improvement will have important impact on the fertility rate after using frozen-thawed semen in ART.

## Supporting information

S1 Table Analysis of variance (ANOVA) of physicochemical characteristics of water. (A) Water size characteristics. (B) Water surface tension characteristics. (C) Water viscosity characteristics. (D) Properties of water density. Refer to text for further details. All p-values adjusted by Bonferroni test; p < 0.001.
(PDF)

S2 Table P-value of pairwise comparison of the effect of different repetition rates factors on physicochemical characteristics. (PDF)

S3 Table P-value of pairwise comparison of the effect of different time periods on physicochemical characteristics. (PDF)

S1 File Minimal data set. Summary physicochemical properties and cellular parameters of sperm association data for all analyses can be found as follows:
(XLSX)

## Abbreviations

ELEFs: extremely low repetition rate electromagnetic fields
TEST: N-tris (hydroxymethyl) methyl-2-aminoethanesulfonic acid + tris
WHO: world health organization
CASA: computer-assisted sperm analysis
DLS: dynamic light scattering
RMS: root mean square
EMF: electromagnetic field
VCL: curvilinear velocity
VSL: straight linear velocity
VAP: average path velocity
LIN: linearity
STR: straightness
ALH: amplitude of lateral head displacement
BCF: beat-cross frequency
HOST: hypo-osmotic swelling test
ROS: reactive oxygen species
MDA: malondialdehyde
TBA: thiobarbituric acid
TCA: trichloroacetic acid
PI: propidium iodide
DCFH-DA: 2′,7′-dichlorofluorescin diacetate
DHE: dihydroethidium
TAC: total antioxidant capacity; DCFH-DA, 2′,7′-dichlorodihydrofluorescein diacetate
DHE: dihydroethidium
PI: propidium iodide
JC-1: 5,5′,6,6′-tetrachloro-1,1′3,3′-tetrathylbenzimidazolyl-carbocyanine iodide
MMP: mitochondrial membrane potential
FITC-PSA: fluorescein conjugated lectin Pisum sativum agglutinin
AO: acridine orange
DFI: DNA fragmentation index
PS: phosphatidlyserine
An: Annexin
Apaf-1: apoptotic protease activating factor 1
ART: assisted reproductive technology
