# Supplementary material for: Electromagnetic field in human sperm cryopreservation improves fertilizing potential of thawed sperm through physicochemical modification of water molecules in freezing medium
Source: PLoS One. 2019 Sep 5;14(9):e0221976. doi: 10.1371/journal.pone.0221976 (PMC6728042; doi:10.1371/journal.pone.0221976)
Supplement: S1 Table — (A) Water size characteristics. (B) Water surface tension characteristics. (C) Water viscosity characteristics. (D) Properties of water density. Refer to text for further details. All p-values adjusted by Bonferroni test; p < 0.001. (PDF) [file pone.0221976.s001.pdf]

Electromagnetic field in human sperm cryopreservation improves fertilizing potential of thawed sperm through physicochemical modification of water Molecules in freezing medium

Dariush Gholami[1,2], Seyed Mahmood Ghaffari[1], Gholamhossein Riazi[1], Rouhollah Fathi[2], James Benson[3], Abdolhossein Shahverdi[2,4*], Mohsen Sharafi[2,5*]

[1]Institute of Biochemistry and Biophysics (IBB), University of Tehran, Tehran, Iran

[2]Department of Embryology at Reproduction Biomedicine Research Center, Royan Institute for Reproductive Biomedicine, ACER, Tehran, Iran

[3]Department of Biology, University of Saskatchewan, Canada.

[4]Reproductive Epidemiology Research Center, Royan Institute for Reproductive Biomedicine, ACECR, Tehran, Iran

[5]Department of Poultry Sciences, Faculty of Agriculture, Tarbiat Modares University, Tehran, Iran

*** Corresponding authors:**

Mohsen Sharafi

Department of Animal science, Tarbiat Modares University (TMU), Tehran, Iran. P.O. Box: 14115-336, Phone No: +98 (021) 48292348. Email: m.sharafi@modares.ac.ir

Abdolhossein Shahverdi

Royan Institute for Reproductive Biomedicine. No.2, Hafez St., Banihashem St., Resalat Ave., Tehran, Iran; P.O.Box: 16635-148; Tel: +98-21-22339940; Fax: +98-21-23562677, Email: shahverdi@royaninstitute.org

S1 Table. Analysis of variance (ANOVA) of physicochemical characteristics of water. (A) Water size characteristics. (B) Water surface tension characteristics. (C) Water viscosity characteristics. (D) Properties of water density. Refer to text for further details. All p-values adjusted by Bonferroni test; $p < 0.001$.

ANOVA table A

Dependent Variable: size (nm)

Source	Type III Sum of Squares	df	Mean Square	F	Sig.
Corrected Model	12412786.962[a]	59	210386.220	3043.559	.000
Repetition rate	7063989.275	11	642180.843	9290.128	.000
time	3577864.398	4	894466.099	12939.821	.000
Repetition rate * time	1770933.290	44	40248.484	582.256	.000
Error	16590.019	240	69.125		
Corrected Total	12429376.981	299			

a. R Squared = .999 (Adjusted R Squared = .998)

ANOVA table B

Dependent Variable: surface tension (mN/m)

Source	Type III Sum of Squares	df	Mean Square	F	Sig.
Corrected Model	3791.828[a]	59	64.268	483.109	.000
Repetition rate	1231.917	11	111.992	841.855	.000
time	2206.995	4	551.749	4147.533	.000
Repetition rate * time	352.916	44	8.021	60.293	.000
Error	15.964	240	.133		
Corrected Total	3807.791	299			

a. R Squared = .996 (Adjusted R Squared = .994)

S1 Table. Continued…

ANOVA table C

Dependent Variable: viscosity (mPa.S)

Source	Type III Sum of Squares	df	Mean Square	F	Sig.
Corrected Model	1.095[a]	59	.019	575.141	.000
Repetition rate	.375	11	.034	1057.303	.000
time	.621	4	.155	4813.474	.000
Repetition rate * time	.098	44	.002	69.297	.000
Error	.004	240	3.226E-5		
Corrected Total	1.098	299			

a. R Squared = .996 (Adjusted R Squared = .995)

ANOVA table D

Dependent Variable: density (g/cm3)

Source	Type III Sum of Squares	df	Mean Square	F	Sig.
Corrected Model	.000[a]	59	7.532E-6	111.296	.000
Repetition rate	.000	11	1.352E-5	199.738	.000
time	.000	4	6.009E-5	887.864	.000
Repetition rate * time	5.535E-5	44	1.258E-6	18.588	.000
Error	8.121E-6	240	6.768E-8		
Corrected Total	.000	299			

a. R Squared = .982 (Adjusted R Squared = .973)
