# Supplementary material for: Electromagnetic field in human sperm cryopreservation improves fertilizing potential of thawed sperm through physicochemical modification of water molecules in freezing medium
Source: PLoS One. 2019 Sep 5;14(9):e0221976. doi: 10.1371/journal.pone.0221976 (PMC6728042; doi:10.1371/journal.pone.0221976)
Supplement: S2 Table — (PDF) [file pone.0221976.s002.pdf]

Electromagnetic field in human sperm cryopreservation improves fertilizing potential of thawed sperm through physicochemical modification of water Molecules in freezing medium

Dariush Gholami[1,2], Seyed Mahmood Ghaffari[1], Gholamhossein Riazi[1], Rouhollah Fathi[2], James Benson[3], Abdolhossein Shahverdi[2,4*], Mohsen Sharafi[2,5*]

[1]Institute of Biochemistry and Biophysics (IBB), University of Tehran, Tehran, Iran

[2]Department of Embryology at Reproduction Biomedicine Research Center, Royan Institute for Reproductive Biomedicine, ACER, Tehran, Iran

[3]Department of Biology, University of Saskatchewan, Canada.

[4]Reproductive Epidemiology Research Center, Royan Institute for Reproductive Biomedicine, ACECR, Tehran, Iran

[5]Department of Poultry Sciences, Faculty of Agriculture, Tarbiat Modares University, Tehran, Iran

*** Corresponding authors:**

Mohsen Sharafi

Department of Animal science, Tarbiat Modares University (TMU), Tehran, Iran. P.O. Box: 14115-336, Phone No: +98 (021) 48292348. Email: m.sharafi@modares.ac.ir

Abdolhossein Shahverdi

Royan Institute for Reproductive Biomedicine. No.2, Hafez St., Banihashem St., Resalat Ave., Tehran, Iran; P.O.Box: 16635-148; Tel: +98-21-22339940; Fax: +98-21-23562677, Email: shahverdi@royaninstitute.org

S2 Table. P-value of pairwise comparison of the effect of different repetition rates factors on physicochemical characteristics.

Pairwise Comparisons A

Dependent Variable: Size (nm)

Repetition rate (Hz)	100	200	300	400	500	600	700	800	900	1000
100	-	P=1.00	P<0.001	P<0.001	P<0.001	P<0.001	P<0.001	P<0.001	P<0.001	P<0.001
200	P=1.00	-	P<0.001	P<0.001	P<0.001	P<0.001	P<0.001	P<0.001	P<0.001	P<0.001
300	P<0.001	P<0.001	-	P<0.001	P<0.001	P<0.001	P<0.001	P<0.001	P<0.001	P<0.001
400	P<0.001	P<0.001	P<0.001	-	P<0.001	P<0.001	P<0.001	P<0.001	P<0.001	P<0.001
500	P<0.001	P<0.001	P<0.001	P<0.001	-	P<0.001	P<0.001	P<0.001	P<0.001	P<0.001
600	P<0.001	P<0.001	P<0.001	P<0.001	P<0.001	-	P<0.001	P<0.001	P<0.001	P<0.001
700	P<0.001	P<0.001	P<0.001	P<0.001	P<0.001	P<0.001	-	P<0.001	P<0.001	P<0.001
800	P<0.001	P<0.001	P<0.001	P<0.001	P<0.001	P<0.001	P<0.001	-	P<0.001	P<0.001
900	P<0.001	P<0.001	P<0.001	P<0.001	P<0.001	P<0.001	P<0.001	P<0.001	-	P<0.001
1000	P<0.001	P<0.001	P<0.001	P<0.001	P<0.001	P<0.001	P<0.001	P<0.001	P<0.001	-

The mean difference is significant at the 0.001 level.

P-value Adjustment for multiple comparisons: Bonferroni

Pairwise Comparisons B

Dependent Variable: Surface Tension (mN/m)

Repetition rate (Hz)	100	200	300	400	500	600	700	800	900	1000
100	-	P<0.001	P<0.001	P<0.001	P<0.001	P<0.001	P<0.001	P<0.001	P<0.001	P<0.001
200	P<0.001	-	P=0.052	P<0.001	P<0.001	P<0.001	P<0.001	P<0.001	P<0.001	P<0.001
300	P<0.001	P=0.052	-	P=0.303	P<0.001	P<0.001	P<0.001	P<0.001	P<0.001	P<0.001
400	P<0.001	P<0.001	P=0.303	-	P=0.217	P<0.001	P<0.001	P<0.001	P<0.001	P<0.001
500	P<0.001	P<0.001	P<0.001	P=0.217	-	P<0.001	P<0.001	P<0.001	P<0.001	P<0.001
600	P<0.001	P<0.001	P<0.001	P<0.001	P<0.001	-	P<0.001	P<0.001	P<0.001	P<0.001
700	P<0.001	P<0.001	P<0.001	P<0.001	P<0.001	P<0.001	-	P<0.001	P<0.001	P<0.001
800	P<0.001	P<0.001	P<0.001	P<0.001	P<0.001	P<0.001	P<0.001	-	P<0.001	P<0.001
900	P<0.001	P<0.001	P<0.001	P<0.001	P<0.001	P<0.001	P<0.001	P<0.001	-	P<0.001
1000	P<0.001	P<0.001	P<0.001	P<0.001	P<0.001	P<0.001	P<0.001	P<0.001	P<0.001	-

The mean difference is significant at the 0.001 level.

P-value Adjustment for multiple comparisons: Bonferroni

Pairwise Comparisons C

Dependent Variable: Viscosity (mPa.S)

Repetition rate (Hz)	100	200	300	400	500	600	700	800	900	1000
100	-	P<0.001	P<0.001	P<0.001	P<0.001	P<0.001	P<0.001	P<0.001	P<0.001	P<0.001
200	P<0.001	-	P<0.001	P<0.001	P<0.001	P<0.001	P<0.001	P<0.001	P<0.001	P<0.001
300	P<0.001	P<0.001	-	P<0.001	P<0.001	P<0.001	P<0.001	P<0.001	P<0.001	P<0.001
400	P<0.001	P<0.001	P<0.001	-	P<0.001	P<0.001	P<0.001	P<0.001	P<0.001	P<0.001
500	P<0.001	P<0.001	P<0.001	P<0.001	-	P<0.001	P<0.001	P<0.001	P<0.001	P<0.001
600	P<0.001	P<0.001	P<0.001	P<0.001	P<0.001	-	P<0.001	P<0.001	P<0.001	P<0.001
700	P<0.001	P<0.001	P<0.001	P<0.001	P<0.001	P<0.001	-	P<0.001	P<0.001	P<0.001
800	P<0.001	P<0.001	P<0.001	P<0.001	P<0.001	P<0.001	P<0.001	-	P<0.001	P<0.001
900	P<0.001	P<0.001	P<0.001	P<0.001	P<0.001	P<0.001	P<0.001	P<0.001	-	P<0.001
1000	P<0.001	P<0.001	P<0.001	P<0.001	P<0.001	P<0.001	P<0.001	P<0.001	P<0.001	-

The mean difference is significant at the 0.001 level.

P-value Adjustment for multiple comparisons: Bonferroni

S2 Table. Continued…

Pairwise Comparisons D										
Dependent Variable: Density (g/cm^3)										
Repetition rate (Hz)	100	200	300	400	500	600	700	800	900	1000
100	-	P=0.875	P<0.001	P<0.001	P<0.001	P<0.001	P<0.001	P<0.001	P<0.001	P<0.001
200	P=0.875	-	P=1.00	P<0.001	P<0.001	P<0.001	P<0.001	P<0.001	P<0.001	P<0.001
300	P<0.001	P=1.00	-	P<0.001	P<0.001	P<0.001	P<0.001	P<0.001	P<0.001	P<0.001
400	P<0.001	P<0.001	P<0.001	-	P<0.001	P<0.001	P<0.001	P<0.001	P<0.001	P<0.001
500	P<0.001	P<0.001	P<0.001	P<0.001	-	P=1.00	P=1.00	P<0.001	P<0.001	P<0.001
600	P<0.001	P<0.001	P<0.001	P<0.001	P=1.00	-	P<0.001	P<0.001	P<0.001	P<0.001
700	P<0.001	P<0.001	P<0.001	P<0.001	P=1.00	P<0.001	-	P<0.001	P<0.001	P<0.001
800	P<0.001	P<0.001	P<0.001	P<0.001	P<0.001	P<0.001	P<0.001	-	P<0.001	P<0.001
900	P<0.001	P<0.001	P<0.001	P<0.001	P<0.001	P<0.001	P<0.001	P<0.001	-	P<0.001
1000	P<0.001	P<0.001	P<0.001	P<0.001	P<0.001	P<0.001	P<0.001	P<0.001	P<0.001	-

The mean difference is significant at the 0.001 level.

P-value Adjustment for multiple comparisons: Bonferroni
