# Supplementary material for: Electromagnetic field in human sperm cryopreservation improves fertilizing potential of thawed sperm through physicochemical modification of water molecules in freezing medium
Source: PLoS One. 2019 Sep 5;14(9):e0221976. doi: 10.1371/journal.pone.0221976 (PMC6728042; doi:10.1371/journal.pone.0221976)
Supplement: S3 Table — (PDF) [file pone.0221976.s003.pdf]

Electromagnetic field in human sperm cryopreservation improves fertilizing potential of thawed sperm through physicochemical modification of water Molecules in freezing medium

Dariush Gholami[1,2], Seyed Mahmood Ghaffari[1], Gholamhossein Riazi[1], Rouhollah Fathi[2], James Benson[3], Abdolhossein Shahverdi[2,4*], Mohsen Sharafi[2,5*]

[1]Institute of Biochemistry and Biophysics (IBB), University of Tehran, Tehran, Iran

[2]Department of Embryology at Reproduction Biomedicine Research Center, Royan Institute for Reproductive Biomedicine, ACER, Tehran, Iran

[3]Department of Biology, University of Saskatchewan, Canada.

[4]Reproductive Epidemiology Research Center, Royan Institute for Reproductive Biomedicine, ACECR, Tehran, Iran

[5]Department of Poultry Sciences, Faculty of Agriculture, Tarbiat Modares University, Tehran, Iran

*** Corresponding authors:**

Mohsen Sharafi

Department of Animal science, Tarbiat Modares University (TMU), Tehran, Iran. P.O. Box: 14115-336, Phone No: +98 (021) 48292348. Email: m.sharafi@modares.ac.ir

Abdolhossein Shahverdi

Royan Institute for Reproductive Biomedicine. No.2, Hafez St., Banihashem St., Resalat Ave., Tehran, Iran; P.O.Box: 16635-148; Tel: +98-21-22339940; Fax: +98-21-23562677, Email: shahverdi@royaninstitute.org

S3 Table. P-value of pairwise comparison of the effect of different time periods on physicochemical characteristics.

**Pairwise Comparisons A**					
Dependent Variable: Size (nm)					
Time (min)	control	15	30	45	60
control	-	P<0.001	P<0.001	P<0.001	P<0.001
15	P<0.001	-	P<0.001	P<0.001	P<0.001
30	P<0.001	P<0.001	-	P<0.001	P<0.001
45	P<0.001	P<0.001	P<0.001	-	P<0.001
60	P<0.001	P<0.001	P<0.001	P<0.001	-
The mean difference is significant at the 0.001 level.<br>P-value Adjustment for multiple comparisons: Bonferroni					

**Pairwise Comparisons B**					
Dependent Variable: Surface Tension (mN/m)					
Time (min)	control	15	30	45	60
control	-	P<0.001	P<0.001	P<0.001	P<0.001
15	P<0.001	-	P<0.001	P<0.001	P<0.001
30	P<0.001	P<0.001	-	P<0.001	P<0.001
45	P<0.001	P<0.001	P<0.001	-	P<0.001
60	P<0.001	P<0.001	P<0.001	P<0.001	-
The mean difference is significant at the 0.001 level.<br>P-value Adjustment for multiple comparisons: Bonferroni					

**Pairwise Comparisons C**					
Dependent Variable: Viscosity (mPa.S)					
Time (min)	control	15	30	45	60
control	-	P<0.001	P<0.001	P<0.001	P<0.001
15	P<0.001	-	P<0.001	P<0.001	P<0.001
30	P<0.001	P<0.001	-	P<0.001	P<0.001
45	P<0.001	P<0.001	P<0.001	-	P<0.001
60	P<0.001	P<0.001	P<0.001	P<0.001	-
The mean difference is significant at the 0.001 level.<br>P-value Adjustment for multiple comparisons: Bonferroni					

**Pairwise Comparisons D**					
Dependent Variable: Density (g/cm^3)					
Time (min)	control	15	30	45	60
control	-	P<0.001	P<0.001	P<0.001	P<0.001
15	P<0.001	-	P<0.001	P<0.001	P<0.001
30	P<0.001	P<0.001	-	P<0.001	P<0.001
45	P<0.001	P<0.001	P<0.001	-	P<0.001
60	P<0.001	P<0.001	P<0.001	P<0.001	-
The mean difference is significant at the 0.001 level.<br>P-value Adjustment for multiple comparisons: Bonferroni					
